# Potential of *in vitro* microelectrode arrays in Alzheimer’s disease research

**DOI:** 10.4103/NRR.NRR-D-24-01582

**Published:** 2025-03-25

**Authors:** Aoife O’Connell, Andrea Kwakowsky

**Affiliations:** Pharmacology and Therapeutics, School of Medicine, Galway Neuroscience Center, University of Galway, Galway, Ireland

Alzheimer’s disease (AD) is a progressive neurodegenerative disorder and is the most prominent cause of dementia. In 2019, over 57.4 million people were living with AD and other dementia subtypes, a number which is expected to increase to over 152.8 million in the next 25 years. This ever-increasing burden has resulted in AD and other neurodegenerative diseases rising to one of the top 10 causes of death globally (O’Connell et al., 2024). The most well-established pathological features of AD include brain shrinkage, accumulation of amyloid-β (Aβ) plaques, neurofibrillary tangles, and a disrupted neuronal network (O’Connell et al., 2024). To date, the cause of AD remains relatively elusive. A plethora of evidence has established Aβ at the forefront of AD pathology, however, the specific mechanisms by which this neurotoxic protein results in the debilitating cognitive decline characteristic of AD remains unknown. It is well-accepted that the excitatory glutamate and cholinergic neurotransmitter systems are severely altered in AD. More recently, evidence has emerged indicating that the disease also affects the inhibitory GABAergic neurotransmitter system (Govindpani et al., 2017; O’Connell et al., 2024). The molecular changes and neuronal loss in these systems disrupt the excitatory/inhibitory balance in the AD brain, potentially contributing to the memory and learning deficits that characterize the condition (Govindpani et al., 2017).

As researchers strive to understand AD pathology, emphasis is often put on the molecular and biochemical changes underlying the condition. However, to fully understand the implications and significance of these changes, focus must also be placed on the functional consequences of these pathological alterations. By investigating the electrogenic features of neurons, the link between cellular and network pathology and disease symptoms can be explored. *In vitro* microelectrode arrays (MEAs) are tools that permit such investigations. They are comprised of grids of tens to thousands of electrodes embedded in a substrate that can record electrical activity noninvasively with a high spatiotemporal resolution. There are various types of *in vitro* MEAs, including the most established planar MEAs, perforated MEAs, which allow increased perfusion, complementary metal-oxide semiconductor MEAs, which have a much higher electrode number, and more recently, three-dimensional MEAs have emerged which improve signal-to-noise ratios (O’Connell et al., 2024). MEAs are very adaptable, permitting the use of various neuronal preperations, including primary cell cultures, acute brain slices, and organotypic brain slices. They can record both spontaneous and evoked responses. Spontaneous activity can be examined by local field potentials (LFP) which are extracellular field changes caused by the summation of ionic processes from all nearby cells. By recording spontaneous LFPs multiple parameters can be investigated depending on the method of analysis used. For example, firing frequency, LFP amplitude, or neuronal bursting provides information on spontaneous activity. By mapping LFP spatial firing patterns and channel cross-correlation, information on network connections can be obtained. MEAs are particularly useful as electrodes not only record nearby electrical signals but also offer the ability to deliver electrical stimuli. This permits experimental replication of physiological processes such as long-term potentiation (LTP) and long-term depression; phenomena which are both strongly linked to learning and memory. The versatile use of MEAs offers a unique ability to assess different neuronal preparations in multiple ways, with just one recording providing a wealth of information. Since MEAs first emerged over 15 years ago several studies have utilized MEAs in the investigation of AD. Our recent review is one of the first to comprehensively examine these studies, presenting the current literature in a way that highlights the potential for MEAs to investigate every aspect of AD from the initial network dysfunction and examining specific pathological features to evaluating new pharmacological approaches for the treatment of the disease (O’Connell et al., 2024).

To date AD neuronal network dysfunction has been investigated using *in vitro* MEAs in three main ways; by looking at synaptic plasticity, network connectivity, and spontaneous activity. Of the eight studies focused on synaptic plasticity highlighted in our recent review, all reported a significant inhibition of LTP in AD models, the primary experimental measure of synaptic plasticity (O’Connell et al., 2024). The majority of these studies modeled AD by acute Aβ treatment (concentrations ranging from 1–5 µM), however, one study used an Aβ intracerebroventricular injection model and three others used transgenic models of AD. Additionally, LTP induction differed between studies with some using a high-frequency and others using a theta-burst stimulation protocol. Despite the methodological differences, all studies reported significantly impaired LTP in each respective model of AD used (O’Connell et al., 2024). This confirms the robust negative effect of AD on synaptic plasticity and highlights the utility of MEAs as an *in vitro* model of the disease with which functional endpoints can be investigated. Fewer studies have investigated AD network connectivity using MEAs, likely due to the need for HD-MEAs, which only became commercially available relatively recently. All studies investigating network connectivity highlighted in our recent review employed primary cell cultures on HD-MEAs, with all reporting a progressive inhibitory effect of Aβ on network connectivity (O’Connell et al., 2024). This once again highlights the substantial negative effect of AD on neuronal networks and represents a useful *in vitro* model for AD. The effects of AD on spontaneous signaling have previously been examined; however, our recent review is one of the first to comprehensively compare each of these MEA-based studies. Unlike synaptic plasticity and network connectivity, a prevailing view of AD’s effect on spontaneous neuronal activity is not as clear. Our review explored each of these studies in detail, giving an overview of what Aβ type, neuronal preparation, and time points were investigated. This detailed comparison helped clarify the inconsistencies previously reported. A particular point of interest is the time points investigated. It appears that the effect of Aβ on spontaneous activity may be a sequential process with initial hyperactivity followed by hypoactivity and that discrepancies in studies are due to the time points investigated. More recent long-term MEA studies support this theory (Varghese et al., 2010; Gao et al., 2019; Liu et al., 2022), as do clinical studies that show neuronal hyperactivity in patients with mild cognitive impairment and neuronal hypoactivity in patients with AD (Busche and Konnerth, 2015).

The use of MEAs in AD research is not limited to investigating neuronal signaling changes, rather they are also valuable tools for examining distinct pathological features of the disease. MEAs have been used to explore the molecular mechanism underlying specific disease features such as dendritic spine loss (Henderson et al., 2019) and intracerebral neuronal communication (Kim et al., 2023). Furthermore, MEAs have been utilized in the investigation of broader pathological aspects including the relationship between AD and type 2 diabetes (Akhtar et al., 2016) and olfactory dysfunction in AD (Liu et al., 2022). The breadth of AD-related pathologies investigated using MEAs underscores their immense potential to advance our understanding of AD pathology. MEAs also represent an underutilized but highly effective approach with which novel therapeutic options may be tested. Our recent work gives a detailed overview of the methodology and the findings of studies utilizing MEAs for this purpose (O’Connell et al., 2024). In these studies, AD models included acute Aβ application or transgenic models. Parameters such as spontaneous signaling or LTP were used to evaluate the effectiveness of the therapeutic in reversing AD pathology. Four studies looked at the impact of altering calcium influx (O’Connell et al., 2024), while others investigated natural compounds such as curcumin, muscone, and ginkgo biloba l. leaf extract among others (Varghese et al., 2010; Bader et al., 2018; Liu et al., 2020). All studies reported promising outcomes; however, the clinical efficacy of these therapies has yet to be established. Nevertheless, these examples of MEAs serving as a functional endpoint with which pharmacological interventions for AD can be evaluated, undoubtedly enhance preclinical evidence.

**Figure 1:**
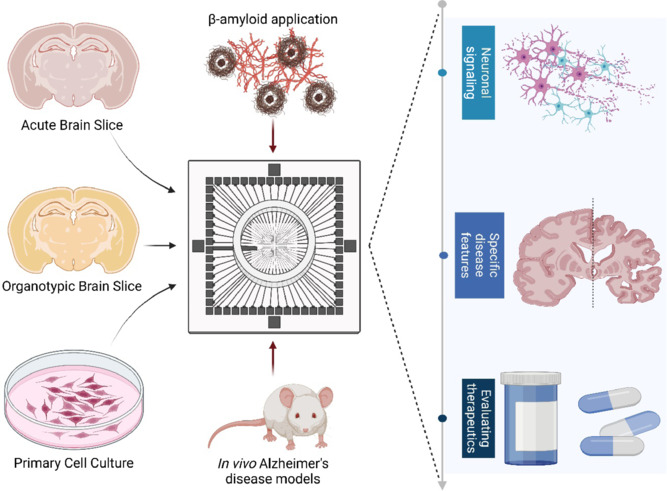
Application of microelectrode arrays in Alzheimer’s disease research. Acute hippocampal slices, organotypic hippocampal slices, and primary neuronal cultures have been used for microelectrode array experiments using Alzheimer’s disease models. This methodology can be applied to all stages of Alzheimer’s disease research, including investigating neuronal signaling and specific disease features and evaluating therapeutics. Created with BioRender.com.

With MEAs, every aspect of AD can be investigated, from basic pathology and specific pathological features to evaluating novel therapeutic options (**Figure 1**). Our recent review provided examples of this in practice at each stage (O’Connell et al., 2024). The effect of AD on neuronal signaling was confirmed in these studies, showing a robust inhibitory effect on synaptic plasticity and neuronal connectivity. The current literature on spontaneous signaling in AD is less conclusive, with inconsistent findings likely due to the versatile use of MEAs leading to methodological differences. However, when interpreted considering these methodological differences, the findings suggest that AD has a sequential effect on spontaneous signaling, initially inducing hyperactivity, followed by hypoactivity. Given their adaptability, MEAs represent an excellent platform for conducting the confirmatory acute and chronic studies required to validate this hypothesis. The flexibility of MEAs compared to traditional electrophysiological methods, makes them an excellent candidate for investigating specific pathological features of AD. MEAs can be applied to any AD model, allowing for targeted investigation of particular disease aspects. Successful examples of this in practice were explored in our recent review, where molecular mechanisms behind pathologies such as dendrite loss and intracerebral neuronal communication were elucidated (O’Connell et al., 2024). MEAs utility in dissecting AD pathology not only helps to identify novel targets for this debilitating disease but also offers a functional model with which novel therapeutics can be tested. Given the high failure rate of AD therapeutics, the addition of this *in vitro* method means biochemical and molecular evidence can be combined with a functional endpoint, improving the preclinical evidence for novel therapeutics.

AD research is inherently complex, as the multifactorial etiology of the condition poses significant challenges both in understanding the cause of AD and in the identification and evaluation of successful therapeutic options. MEAs have consistently demonstrated their effectiveness and value when utilized across various stages of AD research. The findings explored in this perspective highlight the success of MEAs in AD research to date and reaffirm MEAs as a cornerstone technology for all stages of AD research.
